# What affects you? A conversation analysis of exploring emotions during reflection sessions in Dutch general practitioner training

**DOI:** 10.3389/fpsyg.2023.1198208

**Published:** 2023-08-21

**Authors:** Marije van Braak, Sven P. C. Schaepkens, Elise van Dolder, Luna K. Dral, Zoey van der Horst, Daan B. Houben, Emma E. Mees

**Affiliations:** ^1^Department of Language, Literature and Communication, Utrecht University, Utrecht, Netherlands; ^2^Department of General Practice, Erasmus Medical Centre, Rotterdam, Netherlands

**Keywords:** emotion, reflection education, inviting emotion talk, conversation analysis (CA), general practice

## Abstract

**Introduction:**

In Dutch training for general practitioners (GPs), reflection on professional practice is key to their training. Such reflection is considered beneficial for professional development, especially when it entails discussing the emotional dimension of practice experiences. In the GP context, invitations to share the emotional side of things, such as “how did that make you feel?” are considered functional; yet, they are also sometimes viewed by participants as ‘grilling’, ‘just too much’ or ‘too intimate’. Put shortly, putting emotions on the table is institutionally embedded in the GP reflection context, but not always straightforward. Thus, we ask: ‘how do teachers and GP residents invite talk about emotions in educational reflection sessions?’.

**Methods:**

In this study, we explored the Dutch phrase ‘raken, geraakt worden’ (being affected) as one interactional practice used to initiate emotion talk. We conducted a conversation analytic collection study of instances of this phenomenon based on 40 video recordings of hour-long ‘reflection sessions’ at the Dutch GP specialty training. During these sessions, approximately ten GPs in training discuss recent experiences from medical practice under supervision of one or two teachers.

**Results:**

We found that participants orientated to the relevance of ‘being affected’ as a topic for discussion. Variations of the form ‘what affects you now?’ may contribute to putting emotions on the table; they can project a stepwise exploration of the emotional dimension of an experience. The ‘what affects you now’, often done in interrogative format doing a noticing, in combination with a request, is a powerful tool to instigate transformative sequences. The form is less effective to put emotions on the table when the topic shift it initiates is not grounded in previously presented personal stakes or displayed emotion.

**Discussion:**

The study’s findings show how detailed interactional analysis of one sequentially structured practice can benefit education and contribute to theory on emotions and reflection. The mobilizing power of ‘what affects you’ can serve institutional purposes by doing topical work in relation to educational aims, while its power can also be deflated when prior talk does not project the relevance of unpacking the emotional dimension of an experience. Its interactional workings may translate to other helping contexts as well.

## 1. Introduction

Reflection on practice is beneficial for the development of medical professionals during and after their training (Sandars, 2009; Schaepkens and Lijster, 2022). Frequently, experiences that come with strong emotions become meaningful for future practice (Sandars, 2009; Marathe and Sen, 2021), and speaking about them in a reflective setting can be valuable and transformative (Holmes, 2010; Peräkylä, 2019). Talking about emotions can change feelings, thinking and (professional) behavior (Sandars, 2009), and the interactional emotion talk can become a vehicle to display transformation of experience (Peräkylä, 2019). Since emotions significantly impact how professionals do their work, creating attentiveness to the emotional dimension among professionals, but also training professionals to talk about the emotional side of their experiences during their medical training, could enrich medical practice (de Carvalho Filho et al., 2020; Ajjawi et al., 2022). Nonetheless, publicly showing and addressing emotions during medical training can be challenging, delicate or even scary interactional business for professionals, teachers and students (e.g., van Braak et al., 2021). For instance, emotions can be discursively treated as involuntary displays of (private) inner states in relation to maintaining control (Edwards, 1999).

Research has shown that in various institutional settings, such as Alcohol Anonymous Groups, elaboration on emotional experiences can be difficult. For instance, AA participants use statements from prior speakers as a resource to ‘find words’ for their own experience (Arminen, 1998). Within the context of the GP specialty training, we found that residents perceive invitations to explore the emotional dimension of experiences during their General Practitioner specialty training sometimes as difficult, grilling, just too much or too intimate (van Braak et al., 2021). Invitations like ‘how did that make you feel?’ can display interest, but they can also be annoying ways to invite reflection on practice experiences (Maltha et al., 2020), or invite residents to merely play along and talk about emotions to pass the course (Birden and Usherwood, 2013; de la Croix and Veen, 2018). Teachers who facilitate reflection on practice must therefore be attentive (Veen and van Braak, 2022), and manage balancing between stimulating professionally meaningful explorations of emotions in a social context with others, while respecting that emotions are personal.

In this study, we will use an interactional approach to study emotions. This approach implies that we understand emotion in talk as performative: people do things with displays of emotion in talk, and any display of emotion should be understood within the specific bounds of its interactional environment (Couper-Kuhlen, 2009; Kupetz, 2014). Consequently, we will not research emotions as an individual’s personal or private experience; rather, emotions are nestled in the interactional activities. We will scrutinize how emotions receive meaning in relation to any preceding talk, while emotional displays simultaneously project follow-up actions in response to the display (Hepburn and Potter, 2012; Peräkylä, 2012; Kupetz, 2014). In short, we will treat emotions as “interactional phenomena, pervasively shaped by the presence of others” (Weatherall and Robles, 2021, p. 3).

We will analyze real-life explorations of emotion during reflection sessions at the Dutch GP specialty training. First, we will provide a brief overview of interactional research on displays and responses to emotions in everyday and institutional settings. Second, we will address how GP teachers and residents are oriented to ‘putting emotions on the table’. Third, we will explore the Dutch phrase ‘raken, geraakt worden’ (being affected) as one particular way how participants put emotions on the table and engage with topicalized emotions. Our research will generate knowledge about the interactional workings of discussing emotional aspects of experiences in an institutional, and specifically an educational context. Moreover, our research will offer an interactional perspective on the role of emotion in reflection in medical education, and our insights will support teachers and GPs in training (residents) when they deal with emotions during medical training. Thus, we ask: ‘how do teachers and GP residents invite talk about emotions in educational reflection sessions?’

While showing emotions and responding to them are everyday interaction business, previous interaction studies show that emotion in talk is complex. For instance, crying “rarely switches on in full form,” but follows after an accumulation of (subtle) distress signals that unfold throughout an interaction, and impact the ongoing talk (Hepburn and Potter, 2012, p. 200). The interactional complexities of distress and responses to distress (e.g., crying, sniffing, silences) are investigated in everyday and institutional interaction analysis (e.g., psychotherapy, see Muntigl and Horvath, 2014; Peräkylä, 2019; also, child protection helpline, see Hepburn, 2004; Hepburn and Potter, 2012). Displays of pain and anxiety are a common theme in medical interaction research (see, e.g., Parry et al., 2019). Also, anxiety-related displays of emotion are investigated in the context of emergency calls (for an overview, see Voutilainen et al., 2010). Research on emotions in various contexts helps to understand its functions and variations. Hepburn (2004), for example, based on child protection helpline interaction, suggests that crying is not really a unified phenomenon in the way that psychologists treat it. Generally, this body of research suggests that emotions are not just private business or displays that mirror internal states; instead, emotions are a complex, rich, social practice (Edwards, 1999), grounded in interaction.

Interaction research scrutinizes how emotional meaning is derived from the delivery of the turn in relation to its preceding turns (Weatherall and Robles, 2021). Turns can carry certain emotional features, such as a heightened energy in terms of intonation, volume, speed, rate (Weiste and Peräkylä, 2013; Weatherall and Robles, 2021), and breathiness (Hepburn and Potter, 2012). Multimodal displays of emotion include, for example, gaze, facial expression and touch (Weatherall and Robles, 2021). “Response cries” (Goffman, 1978) can display surprise, disappointment, or empathy (e.g., Heritage, 2011; Weatherall and Robles, 2021), and particular lexical choices and grammatical structures may all signal emotion (Peräkylä, 2019). In short, emotional features contribute to the rich ascription of emotion in the context of the turn (Stivers and Rossano, 2010; Muntigl and Horvath, 2014). For instance, breathy voice may signal emotion at one point in interaction, but signal out-of-breath-ness in the next. Within the Dutch GP specialty training settings, displays of emotional signals are a common occurrence in its institutional reflective settings (van Braak et al., 2021).

Displays of emotion have certain “mobilizing features” (Muntigl and Horvath, 2014, p. 106) that invite receipts or responses from others. Previous research explored how people respond to emotional displays; for instance, by showing empathy or sympathy (e.g., Heritage, 2011; Ford and Hepburn, 2021). Kupetz (2014) researched facial expressions and follow-up questions in everyday interactions that present candidate understandings of the displayed emotion. These include expressions with mental verbs (“I can understand that”), second stories (Arminen, 2004), and formulations. From conversation analyses of therapy settings, we know that the latter practice is common. Therapists invite patients to elaborate on the emotional side of their experience by formulating an interpretation of yet unshared but noticeable emotional aspects of experience tellings (Muntigl et al., 2014; Muntigl and Horvath, 2014). Formulations about emotional states (Heritage and Watson, 1979; Knol et al., 2020) can topicalize an emotional aspect that is inferable from the client’s prior turns (Muntigl and Horvath, 2014; Knol et al., 2020). Related to formulations are noticings; these name or verbalize previously non-verbalized displays of emotion, such as “I can see some sadness in your eyes, right” (Muntigl and Horvath, 2014, p. 90), and ‘do recognition’ (Voutilainen et al., 2010) of a displayed emotion. In psychotherapeutic settings, the practice of noticing contributes to the general institutional task at hand, “by projecting a sequence that initiates and enables the step-wise entry into exploration” (Muntigl and Horvath, 2014, p. 106).

The aforementioned practices regarding emotion talk make elaboration about the display of emotion appropriate, but responding to emotion displays is not always straightforward. An uptake “involves orienting to something that is displayed (…), rather than to an action, claim or proposition (Hepburn and Potter, 2012, p. 208). Responses to emotional displays can therefore work in various ways. They can disrupt the progression of the interactional activity by inciting crying that prevents further talking, while they can also progress the interaction and create space to explore emotions (Hepburn and Potter, 2012; Muntigl and Horvath, 2014). Uptakes that are relatively implicit, like ‘low-inference’ responses that acknowledge the emotional valence of a client’s turn, thanking someone for sharing an emotional story (Peräkylä, 2019), commiseration (Peräkylä, 2019), ‘take-your-times’ (Hepburn and Potter, 2007; Knol et al., 2020), and imperatives that direct the client to extend their emotional display (e.g., crying; Muntigl, 2020), create interactional slots for putting emotions on the table. More explicit ways of progressing emotion talk have been analyzed by Muntigl et al. (2014) in the context of therapy. They address the therapist’s eliciting practices that prompt clients to formulate the emotional impact (e.g., ‘How did that make you feel?’). By not engaging with the displayed affect, such elicitations are less affiliative than, for example, noticings, but they still can “create an implication that there is more to the client’s story than was said” (Muntigl et al., 2014, p. 757).

Finally, empathic responses function differently in everyday versus institutional contexts, but also between institutional contexts (Hepburn and Potter, 2012; Ford and Hepburn, 2021). Acknowledgements of distress or difficulty, such as ‘It’s frustrating, isn’t it’ can initiate talk beyond the aims of ongoing institutional business in some settings, while it can be central to the institutional business in other settings. In therapeutic contexts, therapists are institutionally entitled to pursue talk on emotionally laden aspects of experiences. They can harness pursuits and explorations of emotion displays to create therapeutical tension, which can be purposefully exploited for therapeutic reasons. Pursuits of emotion, however, are potentially face-threatening acts in contexts where they are farther away from the institutional business. In that case, the person who is invited to share their emotions is at risk of “being too exposed or vulnerable” (Muntigl, 2020, p. 3).

Conversation analytic research on emotion talk in educational settings is quite limited. In educational literature, as well as in medical educational literature, we do see conceptual and empirical claims that talking about emotions is important. Preschool teachers, for example, are encouraged to model talking about one’s emotion by explaining, questioning, or guiding children to use emotion words to convey their emotional stance toward what is happening around them (Yelinek and Stoltzfus Grady, 2019; see also Spilt et al., 2021). Studies like these quite often report observational data that gloss over ways in which teachers and children display emotion in talk. In medical education, consensus is that emotion plays a crucial role in the professional formation of health professionals (McNaughton, 2013), although talking about emotions (while sharing experiences with patients, for example) is often not yet part and parcel of medical training (Shapiro, 2011; McNaughton, 2013; Rydén Gramner and Wiggins, 2020). An interactional perspective on initiating talk about emotions related to professional experiences is clearly lacking (Rydén Gramner, 2022). One exception is work on the enactment of an embodied affective stance in a fiction seminar that was part of medical training. Rydén Gramner and Wiggins (2020) show how a medical student embodies her affective stance; in this case, the telling that includes the enactment just goes on without additional requests for clarification by the listeners. That is, no explicit interactional work (except for listener tokens) is required to keep the emotion ‘on the table’. As such, the study does not provide insight into means to initiate emotion talk if it is deemed relevant but not topicalized by the one telling about an emotion-relevant experience. It also does not shed light on pursuing emotion talk once the emotion is ‘on the table’. These two aspects are particularly relevant in the setting that we will focus on in this paper: reflection sessions between GPs in training. In this setting, there is a precarious line between what is not considered functional to discuss in this medical training setting, and what is. If we would be able to describe how emotion talk is initiated in an institutional setting with specific educational goals, we would therefore not only augment our theoretical knowledge about the form and function of emotion displays in interaction, but will also be able to suggest ways forward to medical educators dealing with the dilemma of putting (and keeping) emotions on the table - or not.

## 2. Methods

### 2.1. Data and participants

**Figure 1:**
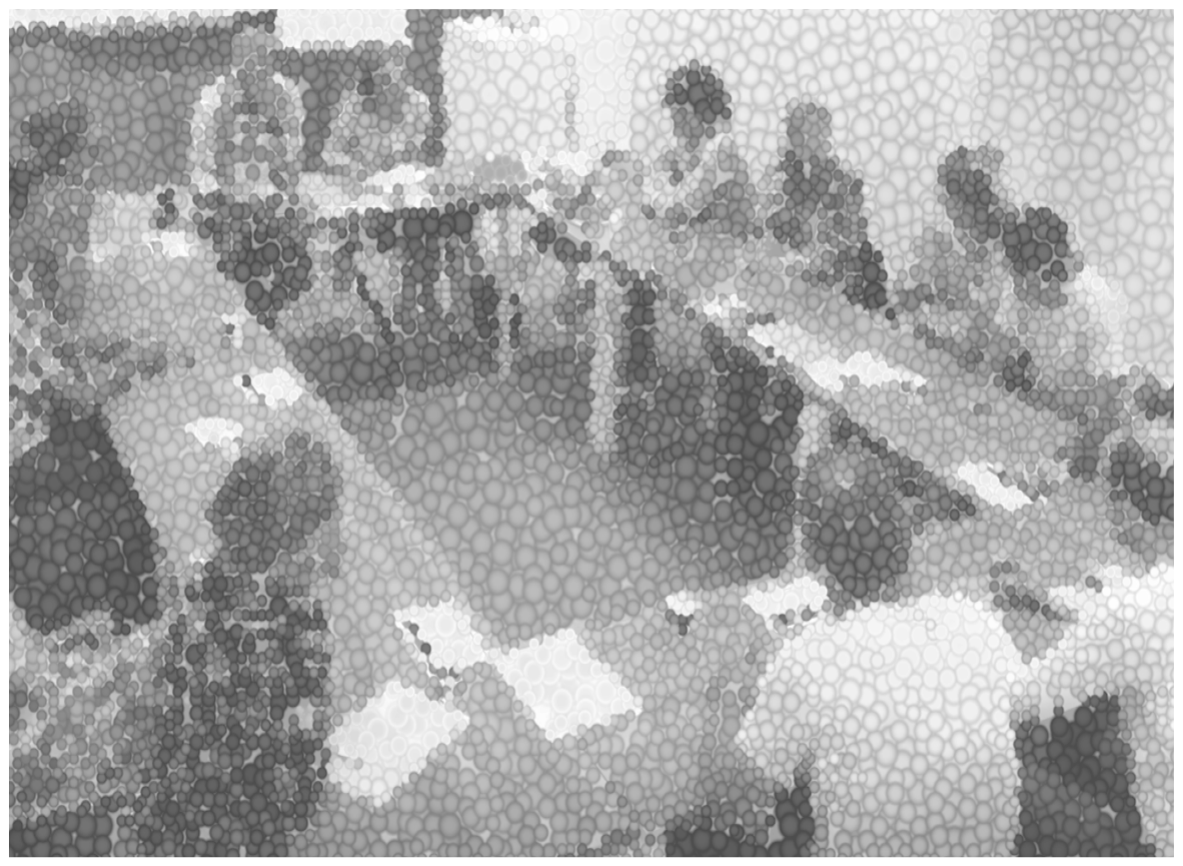
Anonymized overview picture of a ‘Learning from Experiences’-session. The teachers are situated at the head of the table (top left of picture); the other participants are GPs in training.

For this analysis, we drew on 40 video recordings of hour-long ‘Learning from Experience’-sessions at all eight Dutch General Practitioners specialty training institutes. These recordings were collected for the project on teacher facilitation of ‘Learning from Experiences’ (van Braak, 2021). The sessions constituted an integral part of Dutch GP training. They were scheduled weekly during training days at the educational institute, and approximately ten GPs in training discussed recent experiences from practice under supervision of one (15 groups) or two (25 groups) teachers. An anonymized overview picture of one session is presented in Figure 1.

Teachers in the recordings were experienced GPs (35), specialist physicians (1) and behavioral scientists/psychologists (29), who supervised 14 first year, 12 s year, and 14 third year groups. Among others, the shared experiences concerned clinical cases, medical questions, training issues, and the interface between professional and personal life. The sessions’ aim was “creating educational value for future practice based on past experiences shared and discussed in the group” (van Braak et al., 2021; Veen and van Braak, 2022). Furthermore, the sessions offered space for trainees to discuss professional norms, which included talking about the effect experiences could have emotionally (van Braak et al., 2021; Rydén Gramner, 2022). The 40 sessions were recorded between 2017 and 2019 with two or three fixed cameras per session. Participants gave informed consent prior to recording; afterwards, they could request (partial) deletion of the recording. The Dutch Association of Medical Education provided ethical permission to conduct this study (NVMO, case number 829).

### 2.2. Analytic procedure

We analyzed the data in a conversation analytic collection study. Conversation analysis (CA) is an analytic approach that originates in sociology (Goodwin and Heritage, 1990). Closely linked to ethnomethodological approaches (ten Have, 2004), CA describes the interactional procedures that people use to do things in daily life. Through interaction, we construct the social world around us, and the conversation analyst attempts to answer what linguistic forms achieve at particular positions in interaction (Sidnell, 2013).

We conducted the analysis in several steps. After data collection, all video recordings were transcribed verbatim. Building on insights from five BA-thesis projects on ‘inviting emotion talk’ in these data (Dral, 2022; Houben, 2022; Mees, 2022; van der Horst, 2022; van Dolder, 2022), MvB and SS organized four data sessions (see Sidnell, 2013) to explore practices that teachers and residents used to invite talk about the emotional side of an experience. Data sessions were based on detailed transcripts following Jefferson conventions and subsequent refinements of those (Hepburn and Bolden, 2013; silences between turns are unmarked if representing a beat of silence, and otherwise represent an absolute measure of silence, see Hepburn and Bolden, p. 61). Sessions were attended by the authors, fellow conversation analysts and interested communication researchers. First, these preliminary analyses provided interactional evidence that teachers in these sessions oriented to the importance of emotion talk for reflection, and that (not) being emotionally affected can be a concern. Second, there was interactional evidence that GP teachers and residents (un)successfully pursue emotional leads in resident stories. In short, we found how teachers and residents must manage the delicacy of how to invite emotional talk. Although we noticed multiple ways to pursue emotional leads, the elicitation that builds on the word ‘raken’ (i.e., being affected) was a particularly salient way in our data that was used to explore displayed emotions. Therefore, MvB and SS focused exclusively on this form, and identified how different sequential positions and turn constructions contribute to the progress of exploring emotions within this educational setting. Our final collection consisted of 13 excerpts containing a form of ‘being affected’. All transcripts are presented in Dutch (gray) and English. Speakers designated with an A are the residents who share their experience in that Excerpt (A1 for Excerpt 1, A2 for Excerpt 2, etc.), speakers designated with a T are teachers, other speakers (B, C, etc.) are co-residents. Each participant is designated with a unique identifier (letter, number added for tellers and teachers).

## 3. Analysis

Participants in Learning from Experience sessions clearly orient to ‘being affected’ as a relevant (or even urgent) topic for discussion. Invitations in the form ‘how does that affect you’ elicit participants to unpack the emotional dimension, for instance explaining why something has ‘affected’ them. We found that, generally, ‘how does that affect you’ initiates a transformative sequence around the emotional dimension of an experience when there is evidence of what we have called a ‘personal emotional stake’ in the inferential substrate (Haugh, 2022) of talk prior to the invitation. Invitations in this form are unlikely to instigate further talk about emotions if the personal emotional stake is missing. In section 3.1, we first show how participants themselves orient to the relevance of ‘being affected’ as a topic for discussion. In section 3.2, we illustrate how invitations that use ‘being affected’ build on displayed personal emotional stakes and create a context for unpacking emotion. In section 3.3, we provide examples wherein such invitations do not build on prior displays of personal emotional stakes and do not initiate further emotion talk.

### 3.1. Participant orientation to ‘being affected’

In this section, we present evidence that participants in the Learning from Experience sessions are interactionally concerned with being ‘affected’. When someone in the session ‘does being affected’, or presents themselves as ‘having been affected’, it is often picked up and proffered as a topic for discussion. Pursuits around signs of someone ‘being affected’ are another piece of evidence suggesting the relevance of ‘being affected’ to participants in Learning from Experience sessions. Such participant orientation on the relevance of discussing ‘being affected’ is visible in Excerpt 1.

**Excerpt 1:** [[M81129EB; 01:04:45] | T1 = teacher, A1 = teller, others are co-residents.

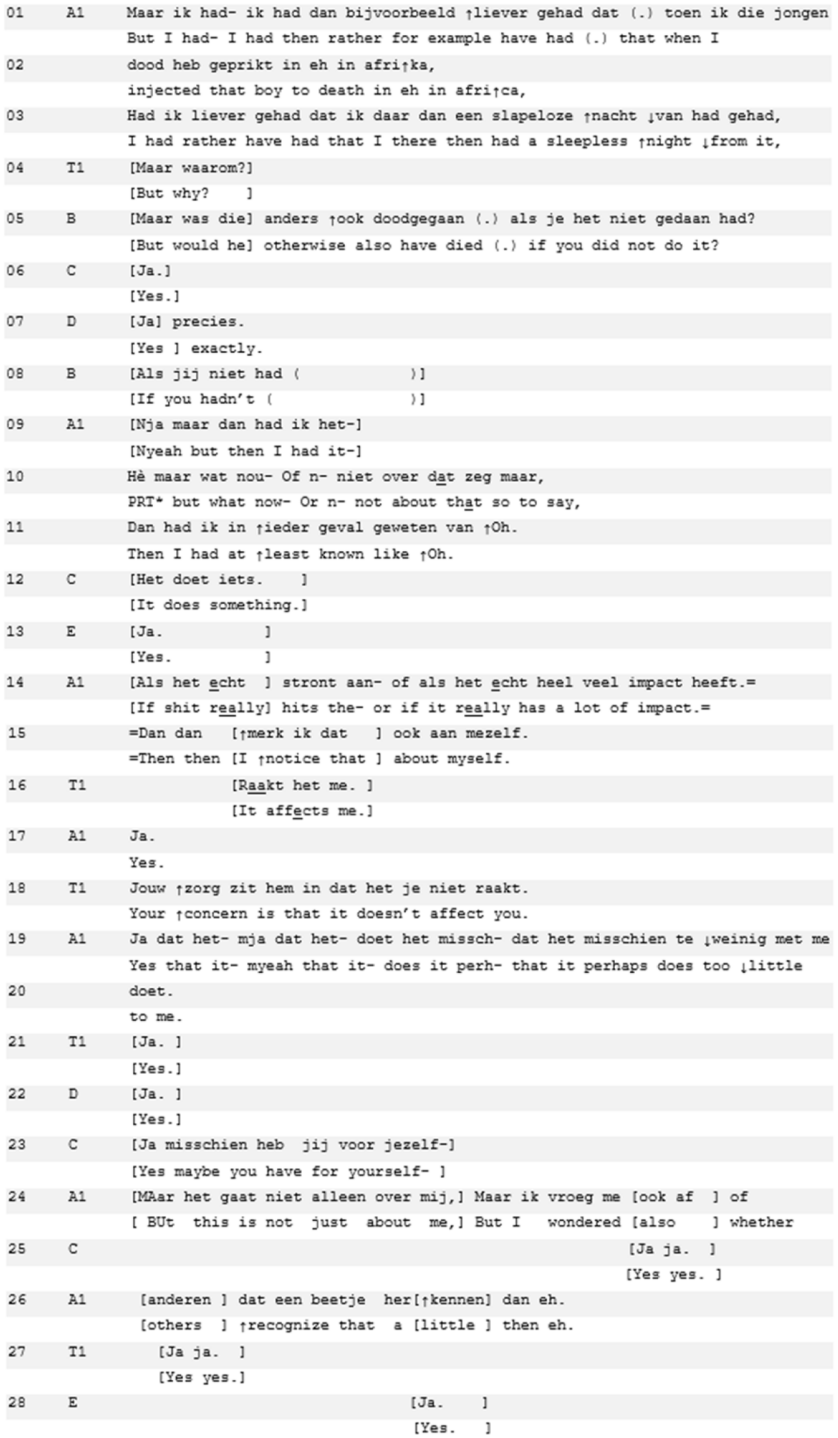

*PRT represents a particle of which no English equivalent exists.

In Excerpt 1, resident A1 presents an experience from his residency in Africa, during which he performed a medical procedure on a patient who soon thereafter passed away. He contrasts the significance of the event with the apparent absence of an emotional response. The teacher’s (T1) uptake of this contrast, using a form of ‘being affected’, is very immediate (line 16–18).

After the resident’s negatively formulated assessment of his reaction to an intense situation (no emotional distress, lines 1–3), the teacher immediately and in overlap formulates (Heritage and Watson, 1979; Knol et al., 2020) the teller’s (A1) concern in terms of (1) being worried and (2) not being affected. This provides a slot for T1’s confirmation (line 16–18). The mention of ‘concern’ in combination with ‘being affected’ draws attention to the personal emotional stake displayed here (*cf.* Flinkfeldt, 2020). The teller shows immediate alignment with this focus (“yes,” lines 17, 19), and upgrades the formulation into not only not being affected, but *unrightfully* being unaffected: “it perhaps does too little to me.” Next, he invites others with a query of recognition, redirecting attention from the topicalized personal stakes to similar experiences of others (lines 24–26). The main takeaway of the quick timing of the teacher’s move toward ‘being affected’, as well as the immediate pick-up for further discussion, is that participants orient to ‘being affected’ as a professional norm and genuine concern (Edwards and Potter, 2017; Kristiansen and Grønkjær, 2018).

With in Excerpt 2, we provide further evidence for the orientation of participants to the relevance of discussing ‘being affected’. Here, an emotional response by resident F to the story of resident A2 becomes part of a procedural negotiation about the session’s proceedings. Resident A2 is telling her experience when resident F interrupts her. She has her arms folded and tears up while talking. With a self-observation that is packaged as a warning, she displays personal emotional stake: “I notice that I feel very strange just now” (not shown). F also displays signs of emotional distress through sudden crying (not shown). F then accounts for her sudden emotional reaction by suggesting she is familiar with A2’s case, knowing the situation privately as an invested bystander (partly displayed, lines 1–6). One resident invites F to expand on her feeling (line 12), while the teacher (T3) proposes to postpone that exploration (lines 14–15):

**Excerpt 2:** [[N81218TFB; 12:18] | T2, T3 = teachers, A2 = teller, others are co-residents.

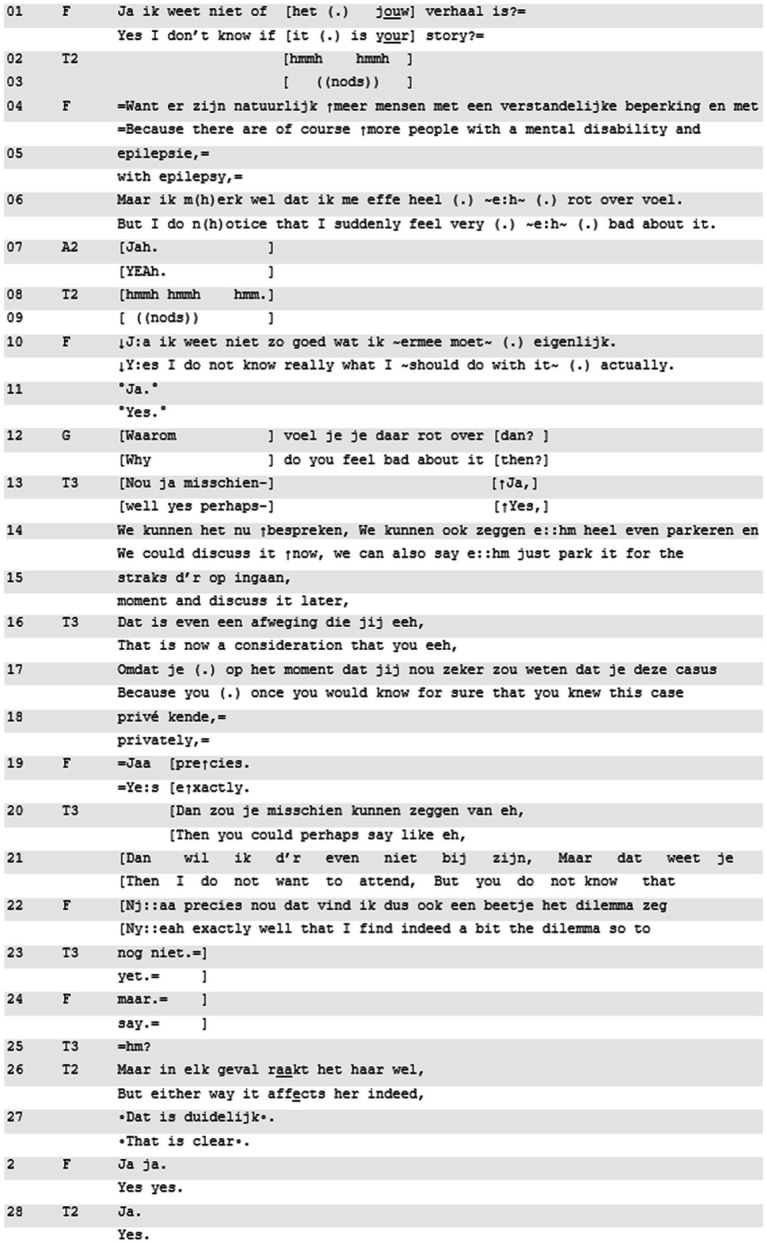

In resident F’s account for her interruption of resident A2’s telling, she leaves the options open as to what will happen next (line 10). However, her turn closings (line 6 and 10), produced partly with shaky voice, mark the importance of her final statement. She foregrounds and intensifies the importance of her sudden and severe emotional reaction. The ambiguity about unpacking the emotional potential of what was just shared, is visible in the two different uptakes (lines 12–15): while one goes along with the unpacking project, the other acknowledges its relevance. Subsequently, two procedural options are proposed: to discuss ‘being affected’ now or later. As Muntigl and Horvath (2014) notice, affectual displays like crying mobilize response, but they do not make the absence of a receipt accountable. Crying may be designed to invite a receipt (Hepburn and Potter, 2007), but does not need to be treated in that way. Thus, the divergent responses are licensed by the production of F’s turn (line 6, 10).

Then, the participants engage in a negotiation about the session’s proceedings. Throughout the interaction, F every now and then wipes tears from her face:

Teacher 3 reformulates the procedural engagement as a moment of “consideration” for resident F (line 16), and offers F the candidate solution to leave the room (“do not want to attend,” in lines 19–21). F remains undecided in her response. Teacher 2’s reaction to that is noticeable, since it does not directly respond to the dilemma that is collaboratively constructed by teacher 3 and F (lines 16–23). Instead, teacher 2 redirects the attention to resident F ‘being affected’. In objective terminology (Potter et al., 2020) and referencing F in the third person, teacher 2 emphasizes the visible urgency of the dilemma (line 25). In a conclusive fashion, teacher 2 notes that F is very affected and “that is clear” for all to perceive. This teacher move explicitly refocuses the interaction toward F’s personal emotional stake, highlighting the need for all participants to do something with the fact that F is visibly affected. Excerpt 2 thus illustrates that ‘being affected’, even if it originally was not the main focus of this Learning from Experience interaction, is topicalized by the resident herself. Furthermore, once it became observable to others, it was attended to by other participants, and started playing an important procedural role in the interaction. Eventually, resident F stayed in the room, but did not join the conversation. After the case discussion was concluded, teacher 2 returned to F and asked “how was this for you to hear?” (not shown). The fact that participants return to the topic after having postponed the matter for quite some time, shows participant orientation to the importance of attending to visible distress.

In sum, Excerpts 1 and 2 provide evidence that residents and teachers explicitly orient to ‘being affected’ as a professional concern that makes further unpacking relevant. When ‘being affected’ becomes visible (Excerpt 1) or topicalized (Excerpt 2), it is treated by the participants as legitimizing instant unpacking, and even temporary abandonment of the primary topic. The excerpts signal that ‘being affected’ is an intricate part of the norms that underlie how reflection sessions are done. In the next sections, we put some flesh on the bones of this orientation on the importance of unpacking ‘being affected’. We first show how explicit invitations with ‘being affected’ are interactionally performed in ways that successfully invite elaboration on the emotional dimension; afterwards, we show how it is less successful.

### 3.2. How invitations with reference to ‘being affected’ invite elaboration

In this section, we show that invitations to explore ‘being affected’ that initiate emotion talk, hinge on the degree to which the invitations build on personal stakes displayed in the inferential substrate of preceding talk (Haugh, 2022). If a resident constructs an experience in terms of personal stakes, the resident highlights emotional commitment and thus creates potential for unpacking the emotional dimensions of the experience. We saw that participants commonly portray their personal stake in one of two ways. First, participants explicitly describe their own relation to a situation in loaded, subjectively invested (Potter et al., 2020) terminology (“difficult,” “personally feel,” “afraid,” “tricky”). Second, participants show further emotional investment through non-verbal displays such as sniffling and creaky voice (Hepburn and Potter, 2012).

In Excerpt 3, we show how the combination of subjectively invested terminology and emotional displays establish a personal emotional stake, which makes attention to it relevant. In preceding talk to the “what affects you now”-invitation that constructs the inferential substrate, resident A3 speaks about having successfully supported a patient in a palliative phase, independently of her supervisor. Nonetheless, she constructs her experience as an instance of being out of place, being “just a youngster,” being inexperienced, and being an intruder (not shown). She foregrounds her subject-position in a situation that is almost too heavy to deal with (partly shown). In doing so, she emphasizes her struggles as a young person taking up responsibility as a doctor (partly shown). Teacher 4 (T4) picks up on A’s existential questions (Haugh, 2022):

**Excerpt 3:** N80516EA; [01:01:50] | T4, T5 = teachers, A3 = teller, others are co-residents

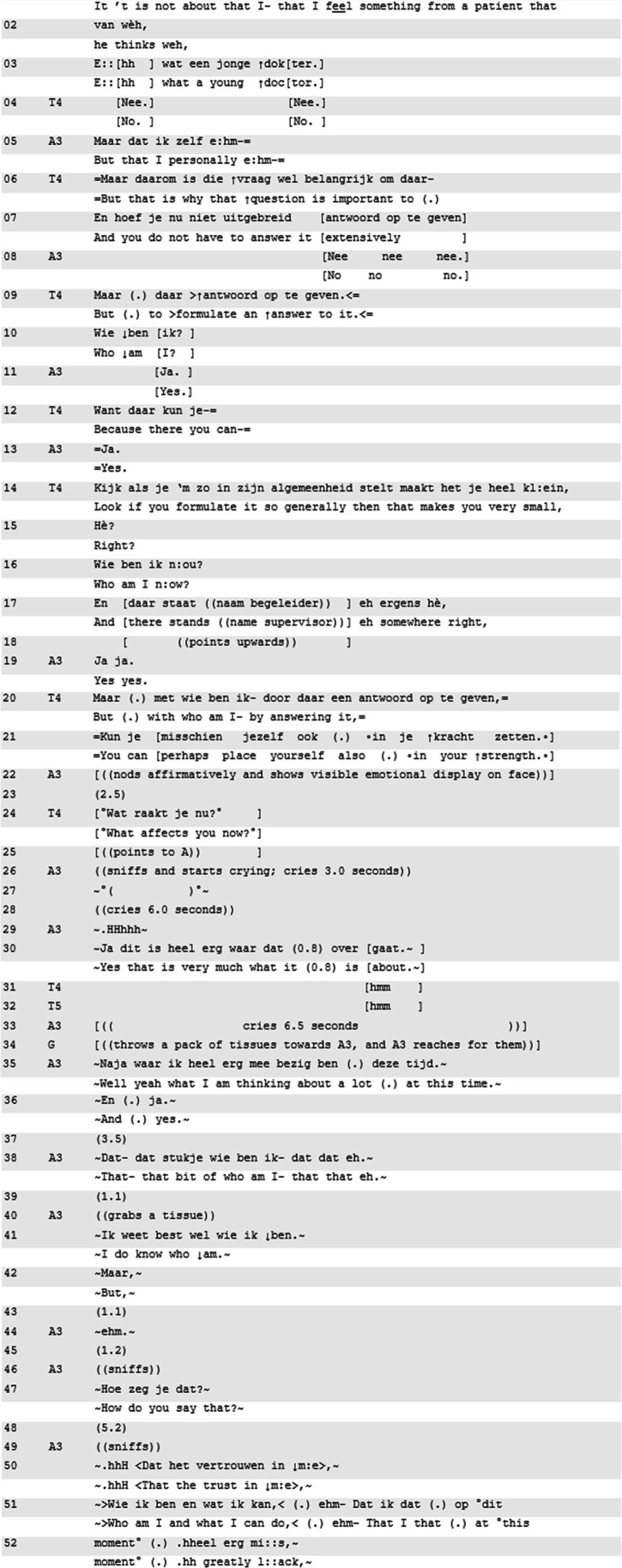

Teacher 4 summarizes A3’s struggles as a central question: “who am I,” and highlights the importance of asking that question (lines 6–21). She works toward formulating a suggestion that may help A3 to find her way in the profession. Up to this point, resident A3 can be seen struggling to hold back her tears, until she starts visibly crying and audibly sniffing (line 26). As Hepburn and Potter (2012) note, sniffing can function as a floor holder, suggesting that the speaker is about to speak but cannot, due to being upset. Indeed, the emotional display prompts teacher 4 to halt her summary, thus allowing for the display to unfold and simultaneously create context for an account of this display.

Crying in itself has been described as doing mobilizing work (Muntigl and Horvath, 2014; Muntigl et al., 2023); crying often gets empathic or sympathetic responses (Heritage, 2011; Ford and Hepburn, 2021). In this case, the crying receipt is done in the form of an invitation to account for the crying, formatted as a “what affects you now” invitation (line 24). This “what affects you now”-formulation topicalizes the emotion, while it is also ‘doing recognition’ of it, as a sign of being affected (Voutilainen et al., 2010). The recognition responds to the disruption of the interaction by making it accountable, which works similarly as crying: “adult crying, and perhaps especially the disruption it causes to the progressivity of sequences, may be accountable (…).” (Hepburn and Potter, 2012, p. 207). While still displaying distress, resident A3 shows alignment with the teacher’s project in her multi-unit turn on identity (“yes, that is very much what this is about,” line 30), and expands on the issue in the following interaction by introducing self-confidence.

In sum, in Excerpt 3 we see how the teacher’s contribution that allows the emotion display to unfold, is co-constructive toward the emotion being topicalized. The “what affects you now”-turn functions in the interactional space as a transformational move (Peräkylä, 2019). During her telling, resident A3 commented on the situation using rhetorical hypotheticals such as “who am I.” The proposed self-deprecating assessments like “I am just a youngster” in prior talk (not shown), are statements that mobilize her personal emotional stake. After the teacher’s invitation in line 24, the topic transforms gradually from a question about “who am I,” which she dismisses by stating that she does know who she is (line 41), to a lack of self-confidence (lines 51–52).

The main takeaways from Excerpt 3 are, first, that the non-verbal emotional distress of the resident was obvious for all to see, and that it was built up from invested personal stakes prior to the “how does that affect you”-invitation. Second, that it was given interactional space to develop. Third, that it got expanded on after the teacher topicalized it in a ‘being affected’-form. With Excerpt 4, we will show how distress can be less obvious, and only noted by some in a second instance. Nonetheless, even small verbal or non-verbal hints of emotional potential after personal stakes have been displayed, can be treated as a discussable or even urgent issue. Similar to Excerpt 3, the resident in Excerpt 4 uses subjectively invested terminology and shows emotional displays in her experience telling. The telling is about an elderly couple that resident A4 spoke to. The man had symptoms that would potentially fit a prostate cancer diagnosis. Over the weekend and prior to the consultation with A4, the couple was misinformed by an unknown GP colleague about the pending protocols to check if the man indeed had prostate cancer.

**Excerpt 4:** [U81016EA; 01:16:26] | A4 = teller, T6 = teacher, others are co-residents.

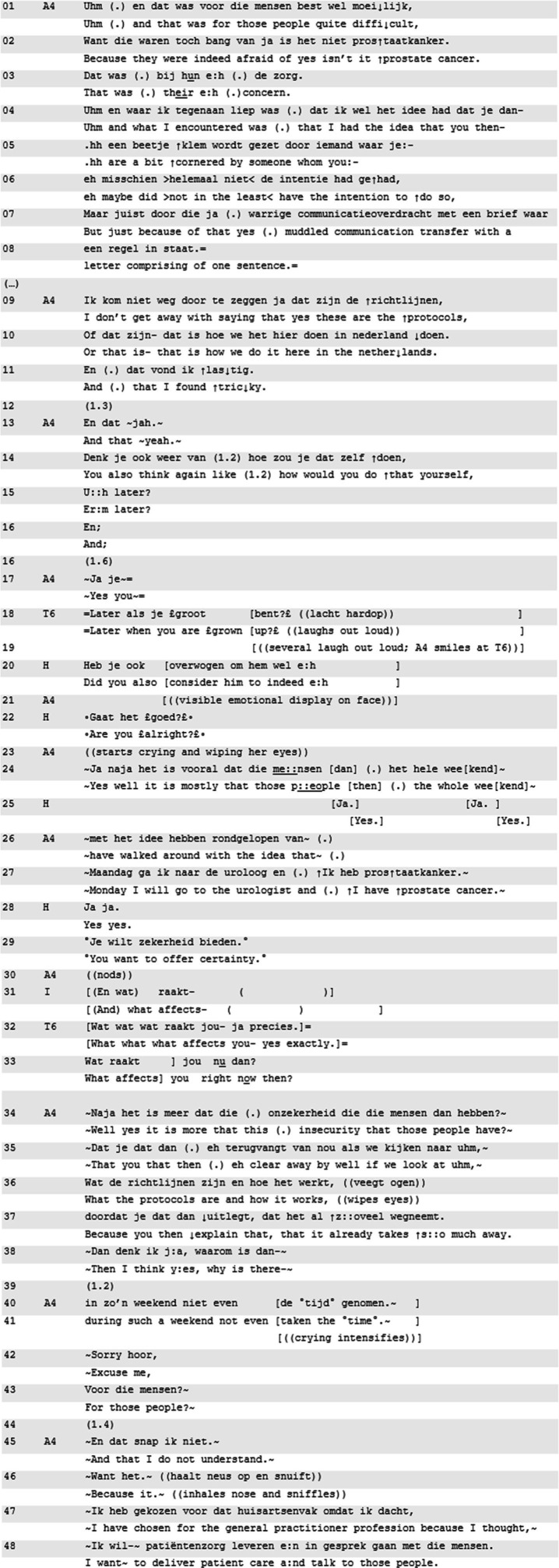

Resident A4 in Excerpt 4 tells the story about how she had to explain procedures and console the two misinformed patients. During the telling, A4 shifts perspectives between the patients’ fear of cancer (line 1), and her own compromised position (lines 4–8), which she assessed as “tricky” (line 11). This is a subject-centered formulation of a troublesome situation that expresses the personal load of the experience. She continues her turn by working toward the relevance of this situation for future practice: “you also think like what would you do yourself in the future” (lines 14–15). The production of this turn is increasingly tremulous and audibly unfinished when she pauses (lines 16–18). Such wobbly delivery can signal emotional or psychological distress (Hepburn, 2004). In fact, the silence, together with the creaky voice, indicates difficulty speaking and implicitly signals upset (Hepburn and Potter, 2012). While the teacher collaboratively constructs A4’s idiom with “later when you have grown up” (line 19), resident H attentively notices A4 struggling to withhold displays of emotion (line 18, 22) by enquiring “are you alright?” (line 23). Formatted as a closed yes/no request for information, it acknowledges the emotion display (Voutilainen et al., 2010), but only minimally invites further elaboration on the visible upset (Hepburn and Potter, 2012). This leaves room for not unpacking it. A4, however, treats it as a request for an elaborate clarification, while also reorienting the perspective from herself to the patients (line 25). It is only when the teacher poses the explicit invitation to explore the emotional dimension (“and what affects you now then?,” lines 35 and further) that the personal involvement of A4 is topicalized and is once more visibly and audibly present. How did this transformation come about?

By using “you” and “now” (line 33), the teacher anchors the observation firmly in the present, and treats the emotional display as something personal and observable (and with that, difficult to circumvent). The teacher acknowledges A4’s emotional stake and provides her with an opening to discuss it. A4 now relates the patient’s distress of being in limbo (line 45) to the re-establishment of her personal stake: “~I have chosen for the general practitioner profession because I thought, I want~ (.) to deliver patient care a:nd talk to those people” (lines 45–47). Her turn is produced in creaky voice and interspersed with sniffles. Thus, the teacher’s “and what affects you now then” functions as a pursuit of the fellow resident’s tentative topicalization of the subtly visible display of emotional distress. This teacher move transforms emotionally laden talk about the situation into explicit discussion of personal emotional stakes (Peräkylä, 2019). Before the teacher’s invitation (line 32–33), A4 referred to a generalized ideal about one’s future professional behavior; after the teacher’s invitation, the referred object transforms into an explicit reference to A4’s own professional identity and the personal choice she made in the past, expressed with lots of displays of emotion throughout.

In sum, with Excerpts 3 and 4 we have shown how residents establish personal emotional stake, often accompanied with emotional displays, and how invitations with ‘how did that affect you (now)’ hook onto the emotional potential that is constructed in the preceding interactional context. First, this move creates an interactional slot for further elaboration of the stakes and emotional dimension. Second, the invitation acknowledges the observable emotional stake, and the participant who is doing the invitation treats the stakes as something important to unpack. The invitations can then incite a transformation of referents (Peräkylä, 2019) that refocuses the interaction from the impersonal and situational to the personal and emotional.

### 3.3. How invitations with reference to ‘being affected’ fail to initiate emotion talk

In this section, we discuss two examples wherein the use of “being affected” did not cause any substantive transformative sequence of personal emotional stake. In both cases, the residents to whom the invitation was posed had not explicated any personal stake through loaded subjective terminology, or shown any emotional displays prior to the invitation. The position and form of the invitation using “being affected” is almost similar to those used in Excerpt 3 and 4; the difference here is the extent to which it is made relevant from the inferential substrate in prior talk (Haugh, 2022).

Excerpt 5 shows a “being affected”-invitation by the teacher, that builds onto a resident story that does not involve an explication of a personal emotional stake in any loaded terminology. Yet, the presented story represents a heavy medical case (a teenager with cancer); its heaviness is recognized in the way other residents respond to the crux of the story. While resident A5 explains how he discovered cancer with a young patient, the other residents in the room respond strongly (lines 10–13, line 21):

**Excerpt 5:** [R80508EC; 29:57] | A5 = teller, T7 and T8 = teachers, others are co-residents.

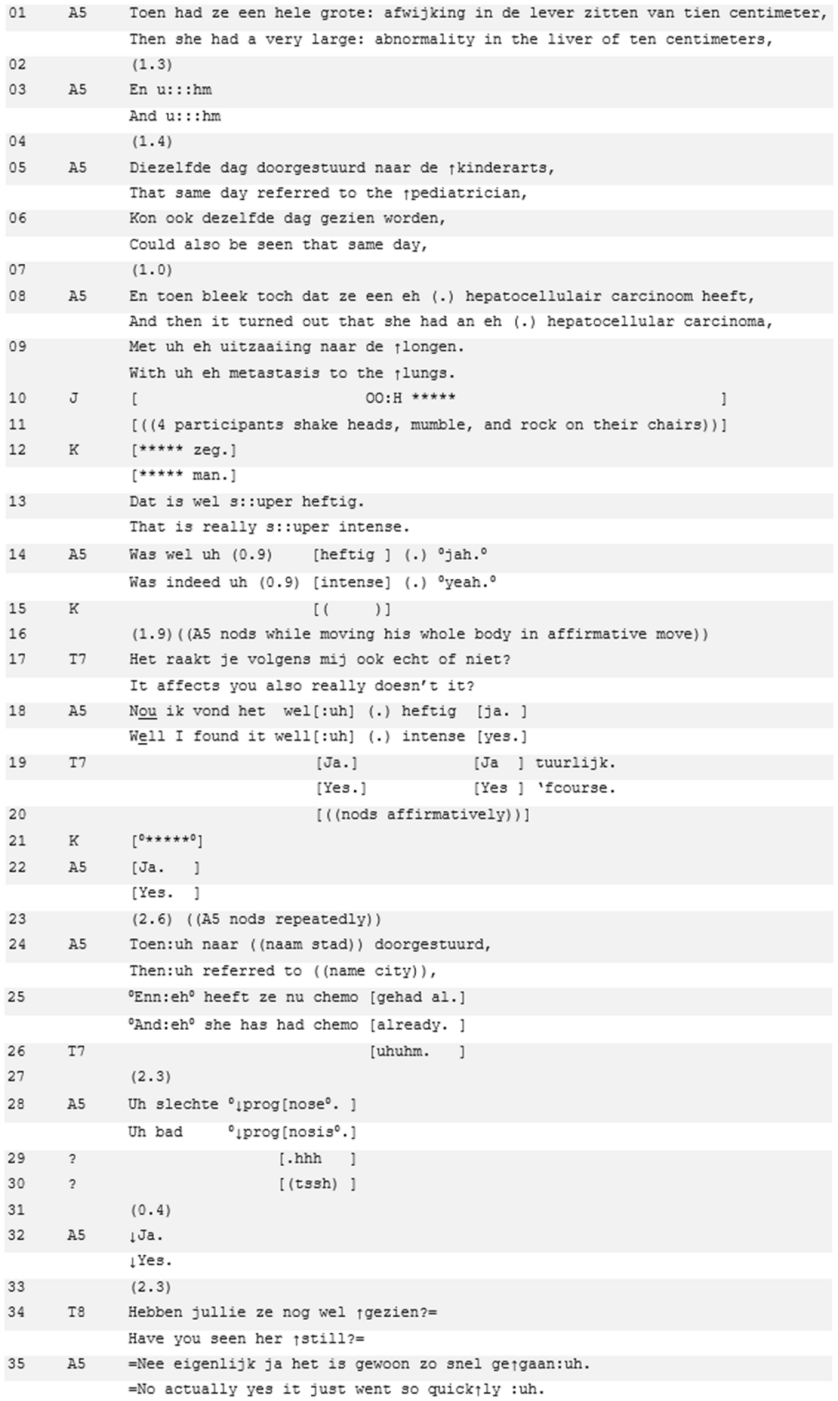

Throughout the case presentation, which is in part visible in lines 01–05, resident A5 solely focuses on the case’s procedural and medical dimension. Thus far, he has not verbally or non-verbally expressed any personal emotional involvement. His speaking manner is remarkably calm (steady rate, even intonation), serious (low intonation, soft speech), and factual (in terms of what happened, and what happened next). The story, however, does elicit several emotionally laden expressions from others (gasping, swear words and extreme case formulation about the intensity of the case, line 10–13, 21). The fellow residents’ intense responses prompt a format tying of “intense” by the telling resident, but he qualifies it in a downgraded way from “su::per intense” to “quite intense” (line 14). The rhythmic gaps in this turn give a sense of slowing down. Though hard to pinpoint, the increasingly slower pace may be what the teacher picks up on when formulating her follow-up invitation, formulated as a noticing (“it affects you also really”; Muntigl and Horvath, 2014) followed by the tag question “does not it?” (line 17).

What happens next is very different from the trajectories following the acknowledgement of ‘being affected’ in Excerpt 3 and 4. Resident A5 acknowledges that it was indeed “quite intense,” again referring to the situation, while explicitly neglecting the invitation to shift toward his personal state that was topicalized in “really affected you.” He continues his story with an orientation to the medical and procedural dimensions of the situation (lines 24–28). The expected outcome of this situation for this patient is interpreted as closure implicative and responded to with soft response cries (Goffman, 1978). Response cries can be seen as emotive interjections (Dingemanse, 2021) that align with the ladenness of an ongoing story (Goffman, 1978); however, no unpacking of any of that emotiveness follows. The first to continue the conversation is teacher 8, who aligns with A5’s procedural focus to elicit further detail on the case specifics: “have you seen her still?” (line 34).

Although it is difficult to definitively conclude that due to the lack of personal stake, the invitation to elaborate on ‘being affected’ did not instigate an exploration of the emotional dimensions, it is markedly missing in Excerpt 5 when compared to Excerpts 3 and 4. Part of this lack of elaboration may also be due to the specific format of the invitation, which is odd in relation to the way the story is told. In telling the story, resident A5 shows no evidence of being affected; however, the teacher refers to him being moved in present tense, suggesting direct evidence in the moment (*cf.* emotional immediacy questions, Muntigl et al., 2023), and using the qualifier “really” to intensify the degree to which the resident was supposedly affected. It is hard to perceive as analysts what elements in the story or the way it was told justify the teacher’s observation. Possibly, the teacher orients to “socially shared expectations regarding emotional experiences and expressions” (Weatherall and Robles, 2021, p. 8; Edwards, 1999). The teacher may act upon the social expectations about the emotional status of someone involved in discovering cancer in a teen, and upon the other participants’ response cries (Goffman, 1978), instead of the emotional stance displayed by the resident (Stevanovic and Peräkylä, 2014). As Stevanovic and Peräkylä note, interactants can use sociocultural resources when interpreting the actions of others, and sometimes these result in a mismatch with the actual emotional stance. Alternatively, the teacher interprets “the objective, distant coolness” of the teller resident as representing his emotional stance; indeed, some could argue that such coolness may signal emotional stance (Stevanovic and Peräkylä, 2014; *cf.* Goffman, 1981; Bakhtin, 1986; Jaffe, 2009; Wilce, 2009). Nonetheless, the link between personal stakes and the role of topicalizing ‘being affected’ in projecting a sequence that initiates stepwise entry into exploration of emotional stance (*cf.* Muntigl and Horvath, 2014) seems deflated here.

Excerpt 6 shows another instance of a teacher reference to ‘being affected’, but it seems unrelated to the presented story and yields little (emotional) alignment. Thus far, resident A6 has shared an experience about a young mentally disabled girl who has been abused by her brother. A6 has not shown any explicit personal stake in loaded terminology or shown any emotional displays, and no unpacking of any emotional dimension ensued. The discussion of this experience is in its concluding phase. As is common in these sessions (Veen and de la Croix, 2017), the teachers formulate a ‘wrap up’ in the form of an evaluation question (lines 8–9). Similar to Excerpt 5, the ‘being affected’-invitation that constitutes this ‘wrap up’ does not instigate any unpacking of emotional stance. Furthermore, once the teacher suggests if the situation has affected resident A6, she explicitly shelves it (lines 10–12).

**Excerpt 6:** M80814DC; 39:00] | A6 = teller, T9 and T10 = teachers.

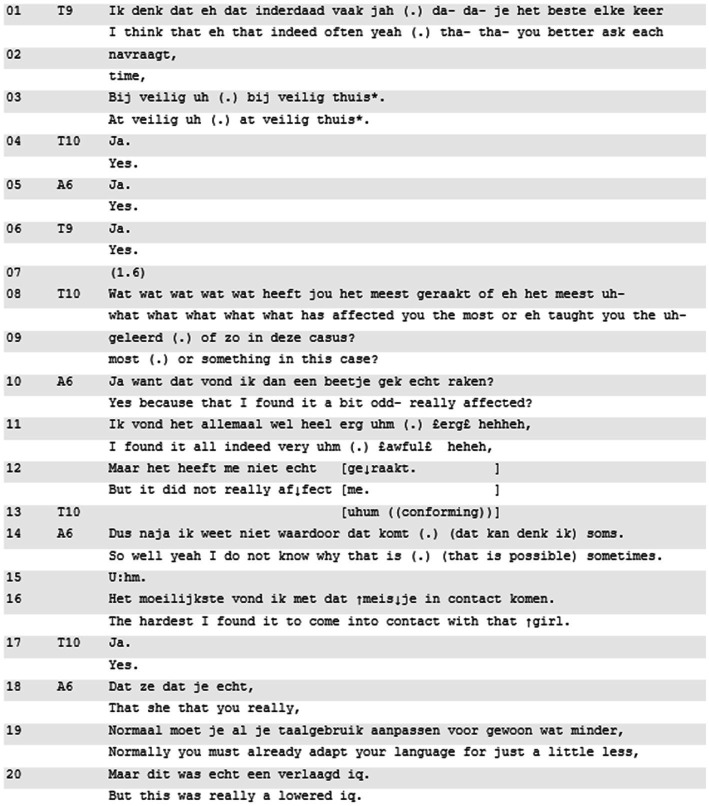

*PRT represents a particle of which no English equivalent exists.

After teacher 9 concludes with the learning uptake (to check some details with Veilig Thuis; line 3), teacher 10 moves into closing with a wrap-up question: “what what what what what has moved you the most or eh (.) taught you the uh most (.) or something in this case?” (lines 8–9). First, what is striking are the many restarts (“what”), the repair (“or uhm”), and the “or something,” which all convey hesitations about the direction of the question. Second, this is a double-barreled question, which constructs a preferred reply to the second part about the learning uptake. Accordingly, resident A6 briefly addresses the first part (lines 10–12), while elaborating on the second (lines 14 and further). In succinctly addressing the inquiry about being affected, A6 disregards the description ‘being affected’ as too heavy a characterization (“really moved?” “did not really move me”). Instead, she comments that she found all of it “a bit odd” because she wasn’t really affected, although the case was quite awful (lines 10–11). This negative topicalization is similar to Excerpt 1: not being affected might also be an issue for residents. Resident A6 then closes the emotional topic by saying she does not “know why that is” (line 14), and replaces the issue of ‘being affected’ with a skills-related topic that was addressed earlier in the interaction (lines 16 and further).

In Excerpt 6, the main takeaway is that the double-barreled question could have allowed for the resident to expand on just the second part of it. However, the resident did not just leave the suggestion of ‘being affected’, instead she explicitly shelved it. There was no explication of any personal emotional stake throughout the case discussion; furthermore, when its presence was suggested, A6 disregarded it as an unjust description of her involvement in the case. The invitation thus remains mostly unsuccessful to elicit any extensive elaboration on why something did (not) affect resident A6. Consequently, there is also no real transformational sequence (Peräkylä, 2019) present regarding A6’s personal emotional stake. This is indicative of the importance of ‘being affected’ to be made relevant for unpacking.

In sum, Excerpt 5 and 6 show how cases that might be considered culturally heavy (and could be potentially emotional), such as the discovery that a young child has cancer or the abuse of a young child by her brother, might not yield substantive explorations of the emotional dimension. It seems that the unsuccessful nature of ‘being affected’-invitations to instigate emotion talk is linked to the lack of an explicated personal emotional stake in loaded terminology, or clearly observable emotional displays during the preceding story telling (Haugh, 2022). There seems to be a mismatch between the probes for emotion and the (lacking) displays of emotional potential that precede those probes.

## 4. Discussion

In this study, we set out to investigate how participants in educational reflection sessions address emotions by focusing on their use of variations on the form ‘what affects you (now)?’. Based on a conversation analytic collection study, we conclude that, first, participants orient to the relevance of ‘being affected’ as a topic for discussion. This orientation was visible in participants topicalizing a lack of being affected (Excerpt 1 and 6), participants readily attending to observable signs of distress (Excerpt 2, 3, 4), even if distress was not the primary focus of the reflection session at that point (Excerpt 2). Second, we conclude that variations of the form ‘what affects you (now)?’ may contribute to putting emotions on the table. They project a stepwise exploration of the emotional dimension of an experience (*cf.* Muntigl and Horvath, 2014). Third, whether the topic is indeed unpacked, seems largely dependent on the extent to which the invitation ‘what affects you (now)?’ is aligned with the emotional potential in the inferential substrate (Haugh, 2022) of talk prior to the ‘what affects you’-intivation (Excerpts 1–4 versus 5 and 6).

Participants’ orientation to ‘being affected’ as relevant for unpacking the emotional potential in interaction may seem counterintuitive, if we compare it to responses to emotional distress in other contexts such as everyday interactions. Responses other than empathic ones may be treated as marked (*cf.* Heritage, 2011). In some institutional interactions, empathic responses could even work against the institutional task at hand. In emergency calls, for example, there is a tension between affiliation and institutional purposes. Here, call takers have been found to address emotion displays mainly in ways that are not affiliative but in function of getting at crucial information (e.g., Kevoe-Feldman, 2021). In the child protection helpline context, a similar tension between affiliation and institutional goals is at play. However, Potter and Hepburn (2010) found more affiliative moves: in child protection helpline interactions, common crying receipts are ‘take your times’ and empathic responses. Hepburn and Potter argue that rather than treating displays of distress as disruptions to the progressivity of the talk, ‘take your times’ and empathic response manage the progressivity of the interaction by allowing its disruption. We see similar openings for emotional disruption to evolve in our data (*cf.* Excerpt 3, 4). Conversely, the directness in timing and form of ‘what affects you?’ in our data, suggests a norm of addressing the observable distress by topicalizing it over affiliating with it.

In a previous study, we found that residents across reflection sessions describe the norm to topicalize emotions as a teacher tendency to ‘dive onto anything emotional’ (van Braak et al., 2021), which they evaluate negatively for its confrontational nature. Confrontations can be regarded as dysfunctional in light of creating a safe space for sharing personal issues (van Braak et al., 2021). In the current study, we see what resistance against such confrontations may look like interactionally. When the ‘what affects you?’-invitation does not build on emotional potential in the form of represented personal stakes and/or displays of emotion (Excerpt 5 and 6) that is sufficiently salient in the inferential substrate of prior sequences (Haugh, 2022), residents tend to not go along with the emotion project. We do not, however, see evidence of obvious resistance to the invitation when it does hook onto prior emotional potential. During earlier phases of this research, we did notice that when probes for emotions were combined with another non-emotional request, residents often responded to the non-emotion related invitation to elaborate. They seize the opportunity to work around the emotion-related topic (see also Excerpt 6). All of this suggests that invitations to engage in emotion talk are potentially face-threatening acts that may be problematic to the progressivity of the institutional task at hand (for a discussion of opposition in relation to institutional goals in therapy setting, see Muntigl et al., 2020). Our observations also relate to work by Edwards (1999). In our data, displays of emotion and picking up on them discursively, harbor some of Edwards’ rhetorical emotion contrasts. For instance, probing after emotional displays with ‘how does that affect you now’, seem to echo the popular opinion that emotions can (involuntarily) ‘leak’ past a controlled outer appearance.

Despite their potentially problematic nature, ‘what affects you?’-invitations are crucial from an institutional perspective. In an educational context wherein exploring the meaning of experiences in people’s lives is the main interactional purpose, the mobilizing power of ‘what affects you?’-invitations, is valuable in topicalizing the emotional dimension of experience. ‘What affects you?’-forms have several features that strongly mobilize response (*cf.* Stivers and Rossano, 2010): they do a request in combination of a noticing (namely someone being affected), they are often done in interrogative format, and with direct eye contact between the resident and teacher. Nevertheless, we see that such invitations are not powerful enough to indeed elicit emotion talk at any point when it is seemingly unrelated to prior talk. The difficulty seems to be in the actual relation between the ongoing and proposed topic. In a sense, our ‘what affects you?’ examples illustrate what Koskinen et al. (2021) call “inherent ambiguity as to the topicality of story-responsive questions” (p. 54). Generally, we know that questions are often used to refocus the topic of ongoing talk (Maynard, 1980; Koskinen et al., 2021). Questions that address an ancillary issue (Jefferson, 1984), may in hindsight introduce a step-wise topic shift, but only if it is picked up and evolved as such in the next turns. If the topic is not developed further, it may be interpreted as a disaffiliative move (Heritage, 2011), which probably explains why residents evaluate seemingly unrelated poking into emotions negatively in terms of relational and procedural effects (*cf.* van Braak et al., 2021). This study therefore highlights the importance of the institutional setting for the way participants deal with emotion talk. Although Rydén Gramner and Wiggins’, 2020 study does show that, in one particular medical training setting, emotion talk is given space once it is ‘on’, earlier CA research does not shed light on the interactional dilemma that may be so particular to the institutional setting of education, and specifically medical education. Certainly, further interactional work on emotion talk in educational settings like those is needed to get a hold on its specific workings for medical educational ends.

Our findings have two important practical implications. First, ‘what affects you?’-forms may be a powerful tool for eliciting emotion talk when the participant recognizably displays emotion or personal stakes in their telling. Second, ‘what affects you?’ seems ineffective for eliciting emotion talk when the topic shift toward emotions is not grounded in presented personal stakes or displayed emotion. In short, this teacher move is not a silver bullet that guarantees residents will explore any emotional dimension. We assume that similar teacher moves (like ‘how did that make you feel?’) will also not work if there is no prior establishment of personal stakes in stories about experiences. Even stories that refer to culturally heavy experiences, but lacking in established personal stakes or emotional displays, seem unlikely to elicit emotion talk after ‘what affects you?’-invitations in our data. Such invitations seem to be treated as unwarranted or misaligned, even when it is constructed in alignment with what came prior, in the form of tag questions and as something heard (Hepburn and Potter, 2007). This raises the question whether ‘what affects you?’-forms that poke for emotion instead of building on it should be avoided. This is an educational question that goes beyond our conversation analytic findings, and needs an answer from a teacher professionalization perspective. Participants’ subtle resistance against such poking, however, in combination with the suggestion that participants in interaction “are continually enacting context, making its relevance available in and through their contributions to the interaction” (Heritage and Clayman, 2011, p. 22), suggests that putting emotions on the table no matter what, is beyond the realm of the currently investigated institutional context.

Theoretically, this work adds insights into emotion work around a specific form of inviting emotion talk (‘what affects you?’) that has not yet been addressed in earlier work thus far (*cf.* Muntigl et al., 2014). It shows how its mobilizing power can serve institutional purposes by doing topical work in relation to educational aims, while it also shows how its power can be deflated when prior talk does not project the relevance of unpacking the emotional dimension of an experience. The work transfers knowledge about conversational practices across institutional domains, thus contributing to our understanding of their uses in context. Despite these contributions, the analytic import of our observations has limitations. We do not have direct empirical evidence for a causal link between the fact that emotion talk does not come about and the lack of a link with prior emotional potential in the invitation. Future research exploring this link in depth, for example by focusing on other forms of invitations that do not seem to instigate emotion talk in this setting, may be a fruitful venue for expanding our understanding of the interactional workings of this one key educational ‘tool’ in reflection sessions: invitations to explore the emotional side of past experiences.

## Data availability statement

The datasets presented in this article are not readily available because of the sensitivity of the data and restrictive informed consent of participants. Requests to access the datasets should be directed to MB, m.vanbraak@uu.nl.

## Ethics statement

The studies involving human participants were reviewed and approved by the Dutch Association of Medical Education (NVMO, case number 829). The patients/participants provided their written informed consent to participate in this study.

## Author contributions

MB collected the data and contributed to the conception of the study. MB, ED, LD, ZH, DH, and EM conducted an initial analysis of the data. MB and SS did the primary analysis for this study and co-wrote a first draft of the manuscript. ED, LD, ZH, DH, and EM were involved in analysis in data sessions. All authors contributed to manuscript revision and approved the submitted version.

## Funding

The data collection for this research was supported by ZonMW, HGOG, research grant number 839130009.

## Conflict of interest

The authors declare that the research was conducted in the absence of any commercial or financial relationships that could be construed as a potential conflict of interest.

## Publisher’s note

All claims expressed in this article are solely those of the authors and do not necessarily represent those of their affiliated organizations, or those of the publisher, the editors and the reviewers. Any product that may be evaluated in this article, or claim that may be made by its manufacturer, is not guaranteed or endorsed by the publisher.
